# Urinary Vitamin D Binding Protein and Kidney Injury Molecule-1 Are Potent Predictors of Acute Kidney Injury After Left Ventricular Assist Device Implantation

**DOI:** 10.3390/biomedicines13112682

**Published:** 2025-10-31

**Authors:** Shiyi Li, Iván Murrieta-Álvarez, Katherine V. Nordick, Zachary Gray, Camila Hochman-Mendez, Alexis E. Shafii, Kenneth K. Liao, Carl P. Walther, Nandan K. Mondal

**Affiliations:** 1 Michael E. DeBakey Department of Surgery, Division of Cardiothoracic Transplantation and Circulatory Support, Baylor College of Medicine, Houston, TX 77030, USA; 2 Department of Regenerative Medicine Research, Texas Heart Institute, Houston, TX 77303, USA; 3 Section of Nephrology, Selzman Institute for Kidney Health, Department of Medicine, Baylor College of Medicine, Houston, TX 77030, USA

**Keywords:** mechanical circulatory support device, acute kidney injury, Vitamin D binding protein, kidney injury molecule-1

## Abstract

**Background/Objectives**: Acute kidney injury (AKI) is a frequent and serious complication following left ventricular assist device (LVAD) implantation. This study aimed to predict AKI within 90 days post-LVAD by evaluating urinary levels of vitamin D binding protein (VDBP) and kidney injury molecule-1 (KIM-1). **Methods**: We prospectively enrolled 29 advanced heart failure patients undergoing LVAD implantation and categorized them into four groups based on pre-LVAD kidney function and hemodynamic stability. Early-morning urine samples were collected 24 h before and 1 week after surgery. Urinary VDBP and KIM-1 levels, normalized to creatinine, were measured. **Results**: Thirteen patients developed AKI postoperatively. Both biomarkers were significantly elevated in patients with pre-existing kidney dysfunction and hemodynamic instability, as well as in those who developed AKI. Pre-LVAD VDBP and KIM-1 levels were associated with a nearly two-fold increased AKI risk. After adjusting for kidney function and hemodynamic status, this risk rose to 776% for KIM-1 and 674% for VDBP. **Conclusions**: Urinary VDBP and KIM-1 are promising non-invasive biomarkers for predicting AKI in LVAD patients. The predictive performance can be greatly improved after combining with pre-LVAD kidney function and hemodynamic stability. Early measurement may help identify high-risk individuals and guide perioperative management.

## 1. Introduction

Left ventricular assist devices (LVADs) serve as a life-sustaining therapy for patients with advanced heart failure, either as a bridge to transplantation (BTT) or as destination therapy (DT). However, this life-sustaining therapy is paradoxically associated with significant post-implantation complications due to high shear stress imposed by LVAD, including bleeding, infection, stroke, and kidney dysfunction [1,2,3,4]. Despite advances in perioperative management and device engineering, acute kidney injury (AKI) remains a common and serious complication following LVAD implantation and is associated with increased morbidity and mortality [5,6,7]. Early identification of high-risk patients and timely implementation of individual interventions may reduce the incidence and severity of postoperative AKI [8,9]. Therefore, the identification of reliable biomarkers that can predict AKI and facilitate perioperative risk stratification is of critical importance in this high-risk population.

Currently, there are several biomarkers, such as cystatin C, neutrophil gelatinase-associated lipocalin (NGAL), IL-18, and tissue inhibitor of metalloproteinase 2 (TIMP-2), that may predict AKI and enable the identification of patients with evidence of kidney injury. In our previous study, we reported that plasma cystatin C can be a promising biomarker for AKI prediction [4]. Sumida et al. reported that measurement of perioperative plasma NGAL can be useful for predicting AKI and renal recovery in LVAD patients [10]. Compared to blood-based biomarkers, urinary biomarkers are considered ideal non-invasive matrices for biomarker assessment, especially for renal diseases whose direct origin is from the kidneys, and it is easy to collect. A previous study by Kiss et al. which reported that urinary IL-18 and TIMP2 can be useful biomarkers for predicting AKI in cardiac surgery patients [11]. IL-18 is a proinflammatory cytokine and lacks specificity; it may be elevated in systemic inflammation or other conditions, and TIMP-2 is more general, which reflects stress rather than specific tubular injury [12]. These limitations underscore the need for tubular injury-specific biomarkers.

Urine vitamin D binding protein (VDBP) is a member of the albumin superfamily of binding proteins that is synthesized in the liver, filtered through the glomerulus, and actively reabsorbed by proximal tubular epithelial cells [13]. When tubular reabsorption capacity is impaired, such as in AKI, urinary excretion of VDBP can increase, making it a potential marker of proximal tubular dysfunction. In one recent study from Diaz-Riera et al., using the proteomic approach to identify early changes in the differential urine protein signature associated with the development of renal injury in acute decompensated heart failure patients, urinary VDBP was identified as the strongest predictor for kidney injury, even before pathological worsening of GFR is evidenced [14]. Moreover, elevated urine VDBP was found to be associated with worse outcomes in patients who develop AKI after cardiac surgery [15]. Urine VDBP may serve as a promising non-invasive biomarker to predict AKI in LVAD patients.

Kidney injury molecule-1 (KIM-1), another kidney damage marker, is a type 1 transmembrane receptor glycoprotein that is expressed on the surface of proximal tubular epithelial cells following kidney injury, which can be detected in urine, making it a highly sensitive and specific biomarker for early tubular injury. In a large prospective cohort study with over 10 years of follow-up, elevated KIM-1 levels were associated with a decline in eGFR, suggesting its potential utility as a predictor of renal function deterioration [16]. Recent investigations have explored the predictive performance of KIM-1 which has shown promise in early AKI prediction and risk stratification [17,18,19]. Furthermore, elevated urinary KIM-1 levels have been implicated in the early detection of allograft rejection-associated AKI in kidney transplant recipients [20]. Despite growing evidence in these clinical contexts, the role of urinary KIM-1 and VDBP in predicting postoperative AKI in LVAD patients remains poorly understood and requires further evaluation. Integrating these biomarkers into perioperative monitoring may provide a translational advantage for early renal risk stratification. KIM-1 elevation reflects proximal tubular injury and repair processes, whereas urinary VDBP loss indicates impaired tubular reabsorption capacity [13,21]. In LVAD recipients, perioperative hemodynamic instability could activate both pathways. Measurement of KIM-1 and VDBP may therefore allow clinicians to identify patients at heightened risk of AKI before serum creatinine rises, enabling early nephroprotective interventions and tailored management strategies. Therefore, this study aimed to evaluate perioperative changes in urinary KIM-1 and VDBP levels and to explore their predictive value for postoperative AKI in LVAD patients, with an emphasis on their potential clinical applicability for early risk prediction and individualized perioperative kidney protection.

## 2. Materials and Methods

### 2.1. Study Design and Groups

The study was conducted at our institution, adhering to the Standards for Reporting of Diagnostic Accuracy guidelines. We enrolled patients who were diagnosed with advanced HF and were scheduled for LVAD implantation prospectively and consecutively. The exclusion criteria included patients who were under 18 years of age, had developed AKI before LVAD implantation, and those who had LVAD implantation but were lost to follow-up visits. The institutional IRB (H-45532) approved the study, and informed consent was obtained from all patients.

The criteria for identifying kidney dysfunction involved a pre-LVAD estimated glomerular filtration rate (eGFR) of less than 60 mL/min/1.73 m^2^, indicating a significant decline in kidney function. Additionally, hemodynamic instability was classified according to the INTERMACS (Interagency Registry for Mechanically Assisted Circulatory Support) profiles 1 and 2, which denote critical clinical states. Based on these parameters, patients were systematically organized into four distinct groups, each reflecting variations in pre-LVAD kidney function and hemodynamic profiles, allowing for a nuanced understanding of their clinical conditions.

- Group 1: normal kidney function (eGFR > 60) with unstable (INTERMACS 1 & 2) hemodynamics (N = 7).
- Group 2: normal kidney function (eGFR > 60) with stable (INTERMACS 3) hemodynamics (N = 10).
- Group 3: impaired kidney function (eGFR < 60) with unstable (INTERMACS 1 & 2) hemodynamics (N = 6).
- Group 4: impaired kidney function (eGFR < 60) with stable (INTERMACS 3) hemodynamics (N = 6).

**Figure 1:**
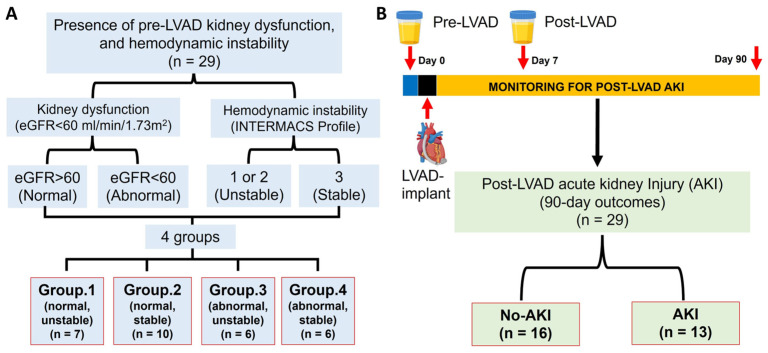
Schematic representations of the study design algorithm. (**A**) Patients are classified based on pre-LVAD kidney dysfunction and hemodynamic instability; (**B**) A timeline for urine sampling and patient grouping by the occurrence of post-LVAD AKI or the absence of it. LVAD stands for left ventricular assist device; eGFR refers to estimated glomerular filtration rate; AKI indicates acute kidney injury; and INTERMACS refers to the interagency registry for mechanically assisted circulatory support.

The early-morning voided urine was collected before (pre-LVAD) and 1 week after LVAD implantation (post-LVAD). The primary endpoint of this study was the development of AKI in the 90 days following LVAD implantation; thus, the patients were grouped according to the presence or absence of AKI as the No-AKI group (N = 16) and the AKI group (N = 13) (Figure 1B). No patient developed AKI during the first postoperative week. Acute kidney injury was defined according to kidney disease: Improving Global Outcomes (KDIGO) guidelines as either of the following: increase in serum creatinine by greater than or equal to 3 mg/dL (≥26.5 μmol/L) within 48 h or need for renal replacement therapy (RRT), or an increase in serum creatinine to greater than or equal to 1.5 times baseline [4,22]. This grouping strategy is visually summarized in Figure 1 to provide a clear overview of the study design.

### 2.2. Biomarkers’ Measurements

Acquired urine samples were centrifuged at 2000× *g* for a period of 15 min, and the supernatant urine samples were aliquoted and kept in 1.5 mL Eppendorf tubes at −80 °C for future analysis. Vitamin D binding protein and KIM-1 levels were measured according to the manufacturer’s instructions using commercially available kits: Vitamin D binding protein (R&D System; Cat No. DVDBP0B) and Kidney Injury Molecule-1 (Ray Biotech; Cat No. ELH-TIM1-1).

### 2.3. Statistical Analysis

Data analyses and graphical presentations were conducted using GraphPad Prism version 9.5.1 (GraphPad Software, Inc., La Jolla, CA, USA) and SAS 9.4 software (SAS Institute, Cary, NC, USA). Continuous variables are reported as mean ± standard deviation (SD) or median (25th, 75th percentiles), while categorical variables are presented as percentages following our previous reported studies [23,24,25,26]. We used repeated measurement analysis of variance or the Mann–Whitney U test to compare continuous data between two groups. Categorical variables were compared using the chi-square test or Fisher’s exact test. A *p*-value of less than 0.05 was considered statistically significant.

## 3. Results

### 3.1. Patient Characteristics Among Those with or Without Pre-LVAD Kidney Dysfunction and/or Hemodynamic Instability

The characteristics of patients with and without pre-LVAD kidney dysfunction and/or hemodynamic instability are summarized in Table 1. The clinical and demographic characteristics of patients stratified by pre-LVAD kidney function and hemodynamic status are summarized in Table 1. Overall, most baseline characteristics were comparable among the four groups. The average patient age across groups was approximately 60 years, and most were Caucasian white males. Although patients in Groups 3 and 4 tended to be taller and heavier than those in Groups 1 and 2, most were classified as overweight, with body mass index (BMI) values exceeding 25 kg/m^2^. Ischemic cardiomyopathy was more prevalent in Groups 2, 3, and 4, whereas Group 1 patients more commonly had non-ischemic cardiomyopathy. All patients demonstrated severely reduced left ventricular ejection fraction (LVEF), with group means below 21%. Notably, the left ventricular internal diameter at end-diastole (LViDd) was significantly larger in Groups 2, 3, and 4 compared to Group 1. Postoperative outcomes showed apparent differences among groups. Group 2 patients with preserved kidney function and stable hemodynamics had the shortest durations of mechanical ventilation, intensive care unit (ICU) stay, and total hospitalization. In contrast, Group 3 patients, characterized by both impaired kidney function and hemodynamic instability, experienced the longest postoperative ventilation times, ICU stays, and hospitalizations.

### 3.2. Patient Characteristics by Development of Post-LVAD AKI

The characteristics of patients with and without post-LVAD AKI are summarized in Table 2. Most baseline variables were comparable between the two groups. The majority of patients in both the AKI and no-AKI groups were Caucasian white males over 60 years of age. The goal of LVAD implantation was DT for nearly all patients, with only one patient in the AKI group receiving the device as BTT. In the no-AKI group, over half of the patients had ischemic cardiomyopathy with an average LVEF of around 21%, whereas in the AKI group, non-ischemic cardiomyopathy was more prevalent, and the average LVEF was below 20%. The proportion of chronic kidney disease (CKD) was comparable between the AKI and the no-AKI groups. INTERMACS profiles tended to be higher in the AKI group, indicating a greater degree of preoperative hemodynamic compromise. Moreover, patients who developed AKI after LVAD implantation had significantly longer total hospital stays compared to those without AKI. Although not statistically significant, the AKI group also exhibited longer postoperative mechanical ventilation times and extended ICU stays.

### 3.3. Biochemistry for Patients with or Without Pre-LVAD Kidney Dysfunction and/or Hemodynamic Instability

**Figure 2:**
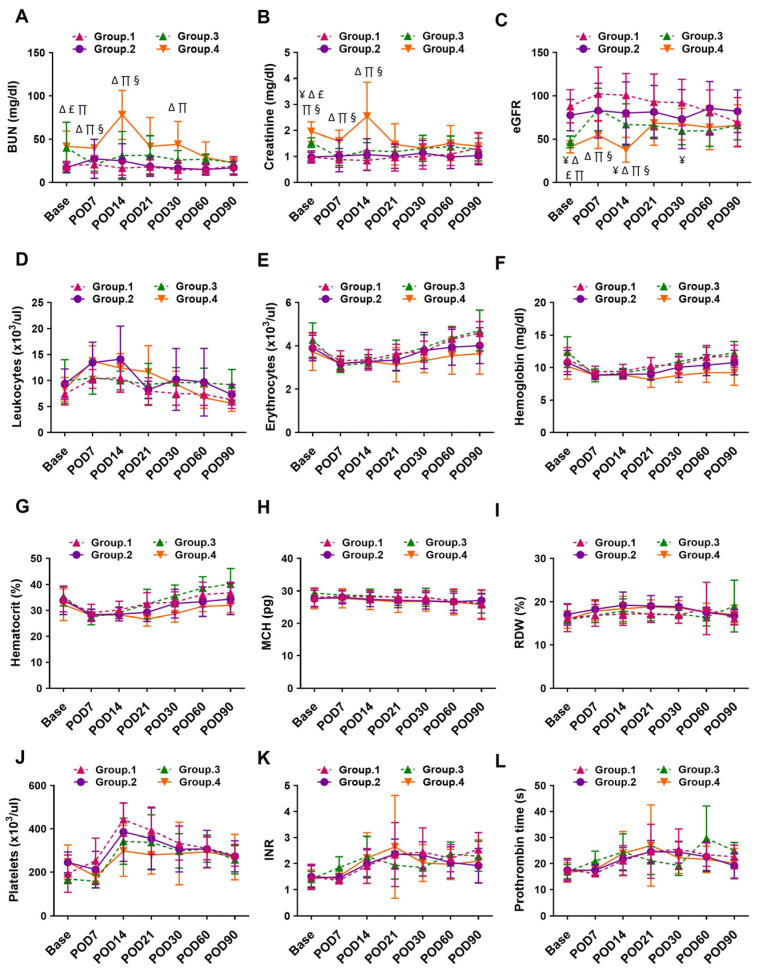
Temporal changes in (**A**) BUN, (**B**) Creatinine, (**C**) eGFR, (**D**) Leukocytes count, (**E**) Erythrocytes count, (**F**) Hemoglobin, (**G**) Hematocrit, (**H**) MCH, (**I**) RDW, (**J**) Platelets count, (**K**) INR and (**L**) Prothrombin time at the baseline and multiple time points after LVAD implantation among 4 groups of patients based on pre-LVAD kidney function and hemodynamic stability. Data are expressed as mean ± SD, *p* < 0.05 is considered significant. ¥: Group 1 vs. Group 3; ∆: Group 1 vs. Group 4; £: Group 2 vs. Group 3; ∏: Group 2 vs. Group 4; §: Group 3 vs. Group 4. BUN, blood urea nitrogen; eGFR, estimated glomerular filtration rate; MCH, mean corpuscular hemoglobin; RDW, red cell distribution width; INR, international normalized ratio; POD, post-operative day.

Routine laboratory data were extracted from the hospital database to investigate hematology and blood chemistry profiles before and multiple postoperative time points up to postoperative day (POD) 90. Pre-LVAD blood urea nitrogen (BUN) and serum creatinine levels were significantly higher in Group 3 and Group 4 patients compared to Group 1 and Group 2, consistent with lower eGFR values observed in Groups 3 and 4. Following LVAD implantation, Group 3 patients exhibited a significant decline in BUN and creatinine within the first postoperative week, which remained stable through POD90. Correspondingly, eGFR increased markedly at POD7, followed by a slight decline, but remained elevated compared to baseline levels throughout the 90-day period. In Group 4 patients, BUN and creatinine levels showed a transient rise at POD14 before gradually declining through POD90. eGFR in this group increased at POD7, decreased at POD14, and subsequently rose significantly, remaining elevated through POD90. In contrast, kidney function parameters (BUN, creatinine, and eGFR) remained generally stable in Group 1 and Group 2 patients throughout the study period. Other hematologic parameters, including leukocyte count and hemoglobin concentration, were comparable across all four groups from baseline to POD90. The routine lab hematology and blood chemistry for four groups of patients based on pre-LVAD kidney function and hemodynamics are presented in Figure 2, and details of mean with standard deviation and *p*-values for renal function profiles are presented in Supplementary Tables S1–S3.

### 3.4. Laboratory Hematology and Blood Chemistry for Patients with or Without Post-LVAD AKI

**Figure 3:**
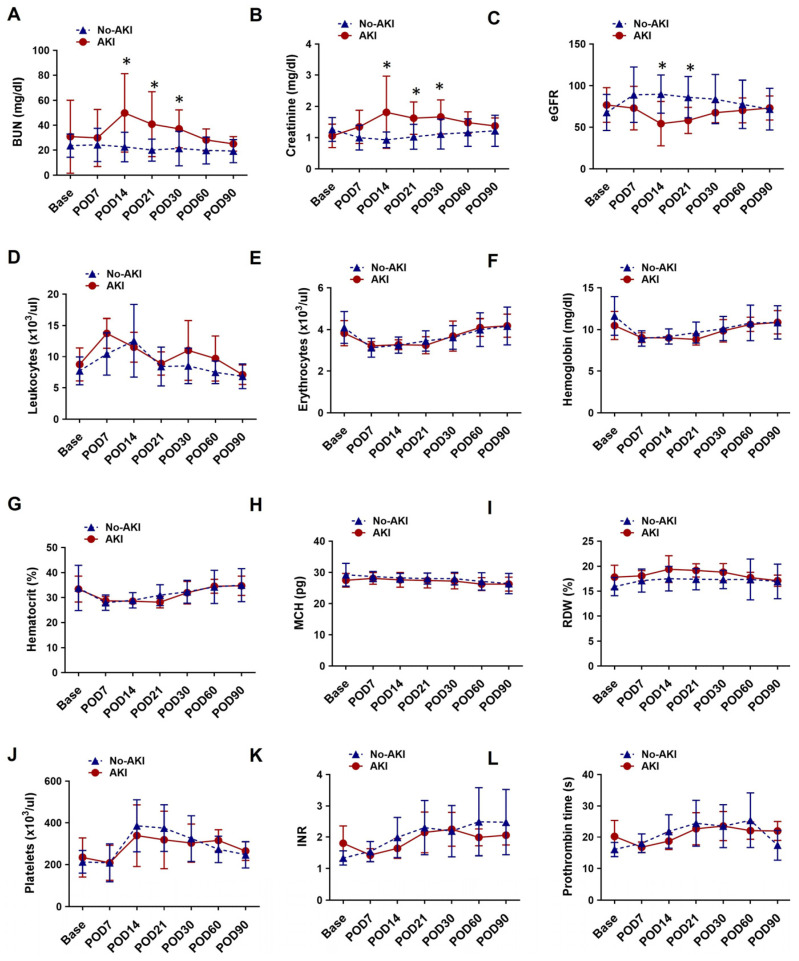
Temporal changes in (**A**) BUN, (**B**) Creatinine, (**C**) eGFR, (**D**) Leukocytes count, (**E**) Erythrocytes count, (**F**) Hemoglobin, (**G**) Hematocrit, (**H**) MCH, (**I**) RDW, (**J**) Platelets count, (**K**) INR and (**L**) Prothrombin time at the baseline and multiple time points after LVAD implantation between AKI group patients and the no-AKI group patients. Data are expressed as mean ± SD, * *p* < 0.05 is considered significant. AKI, acute kidney injury; BUN, blood urea nitrogen; eGFR, estimated glomerular filtration rate; MCH, mean corpuscular hemoglobin; RDW, red cell distribution width; INR, international normalized ratio; POD, post-operative day.

Comparative analysis of laboratory hematology and blood chemistry profiles revealed distinct differences in kidney function trajectories between patients with and without AKI following LVAD implantation. At baseline, BUN, serum creatinine, and eGFR values were comparable between the AKI and no-AKI groups. In the AKI group, BUN and creatinine levels increased significantly after POD 14, followed by a gradual decline toward POD 90. eGFR in this group decreased markedly at POD14, then showed a modest recovery, returning to near-baseline levels by POD90. In contrast, patients in the no-AKI group exhibited a slight decline in BUN and creatinine at POD7, followed by a gradual rise that returned to baseline by POD90. eGFR in the no-AKI group significantly increased at POD7, then steadily declined toward baseline over the remainder of the 90-day period. Other hematologic and biochemical parameters, including leukocyte count and hemoglobin concentration, remained comparable between the AKI and no-AKI groups across all time points. The dynamic changes in routine laboratory kidney function tests and blood profiles are presented in Figure 3, and details of mean with standard deviation and *p*-values for renal function profiles are presented in Supplementary Tables S1–S3.

### 3.5. Change in Urinary VDBP, KIM-1, and Their Correlation with Creatinine-Based Estimated Glomerular Filtration Rate

**Figure 4:**
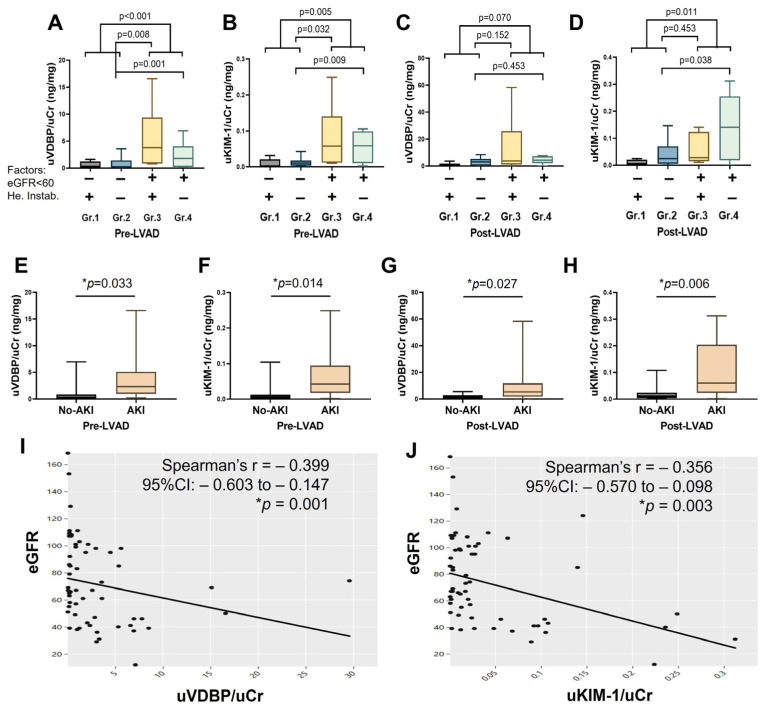
Box-and-whisker plots showing urinary biomarker levels before (pre-) and after (post-) LVAD implantation. (**A**,**B**) Pre-LVAD urinary VDBP and KIM-1 (normalized to urinary creatinine) across four patient groups categorized by baseline kidney function and hemodynamic stability. (**C**,**D**) Post-LVAD urinary VDBP and KIM-1 levels among the same four groups. (**E**,**F**) Pre-LVAD urinary VDBP and KIM-1 levels in patients with and without postoperative AKI. (**G**,**H**) Post-LVAD urinary VDBP and KIM-1 levels in AKI versus no-AKI groups. (**I**,**J**) Spearman’s rank correlation between urinary VDBP, KIM-1 (normalized to urinary creatinine), and eGFR. Data are expressed as mean ± SD, * *p* < 0.05 is considered significant. AKI, acute kidney injury; uVDBP, urinary Vitamin D binding protein; uKIM-1, urinary kidney injury molecule-1; uCr, urinary creatinine; CI, confidence intervals; eGFR, estimated glomerular filtration rate; Group 1 (Gr.1), normal kidney function with unstable hemodynamics; Group 2 (Gr.2), normal kidney function with stable hemodynamics; Group 3 (Gr.3), impaired kidney function with unstable hemodynamics; Group 4 (Gr.4), impaired kidney function with stable hemodynamics.

Routine pre-LVAD urinary levels of VDBP and KIM-1 were significantly elevated in patients with impaired kidney function (Groups 3 and 4) compared to those with normal kidney function (Groups 1 and 2) (Figure 4A,B). Moreover, patients in Groups 3 and 4 demonstrated markedly higher urinary VDBP and KIM-1 levels compared to Group 2 patients with normal renal function and stable hemodynamics. Following LVAD implantation, urinary VDBP and KIM-1 levels remained significantly elevated in Groups 3 and 4 when compared to Groups 1 and 2 (Figure 4C,D). While Group 3 and Group 4 patients exhibited relatively higher urinary VDBP than Group 2, these differences did not reach statistical significance (Figure 4C). However, urinary KIM-1 levels were significantly elevated in Group 4 compared to Group 2. No significant difference in post-LVAD urinary KIM-1 was observed between Groups 2 and 3 (Figure 4D).

Among patients stratified by AKI status, both urinary VDBP and KIM-1 levels were significantly higher in the AKI group at baseline (Figure 4E,F). This difference persisted throughout the postoperative period, with AKI patients maintaining significantly elevated urinary biomarker levels compared to the no-AKI group (Figure 4G,H). Spearman correlation reveals both urinary VDBP and KIM-1 significantly negatively correlated with eGFR, with rho = −0.399 and *p* = 0.001 for urinary VDBP and rho = −0.356 and *p* = 0.003 for urinary KIM-1 (Figure 4I,J). Details of the median with IQR and *p*-value for urinary biomarkers are presented in Supplementary Tables S1–S3.

### 3.6. Association of Urinary VDBP and KIM-1 with Post-LVAD AKI

Patients with elevated pre-LVAD urinary KIM-1 and VDBP levels (defined as values two-fold higher than the group median) had 98% and 99% higher odds, respectively, of developing postoperative AKI compared to those with lower pre-LVAD levels. Following LVAD implantation, increased urinary KIM-1 and VDBP levels were also associated with 39% and 26% higher odds of AKI, respectively. These associations were more pronounced among patients with baseline kidney dysfunction and hemodynamic instability. The association between urinary VDBP, KIM-1, and adjusted for pre-LVAD kidney function and hemodynamic stability, and postoperative AKI is presented in Table 3.

### 3.7. Urinary VDBP and KIM-1 for Post-LVAD AKI Prediction

**Figure 5:**
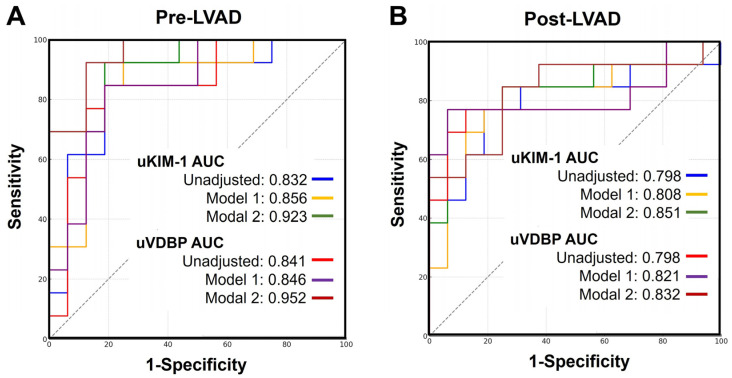
ROC curves of the pre-LVAD urinary VDBP, KIM-1, and adjusted clinical models (**A**) and post-LVAD urinary VDBP, KIM-1, and adjusted clinical models (**B**) for predicting AKI within 90 days after LVAD implantation. AKI, acute kidney injury; LVAD, left ventricular assist device; ROC, receiver operating characteristic.

Both pre-LVAD and post-LVAD urinary levels of VDBP and KIM-1 were evaluated for their ability to predict postoperative AKI within 90 days following LVAD implantation. Individually, pre-LVAD urinary VDBP and KIM-1 demonstrated strong predictive performance, with area under the receiver operating characteristic (ROC) curves (AUC) of 0.841 and 0.832, respectively. Each marker yielded a sensitivity of 84.6% and a specificity of 81.2%. However, the predictive accuracy of post-LVAD urinary VDBP and KIM-1 was slightly reduced, with AUC values of 0.798 and a sensitivity of 76.9% for both markers. Notably, after adjusting for pre-LVAD kidney function and hemodynamic status, the predictive performance of both pre- and post-LVAD biomarkers improved substantially, with increases observed in AUC, sensitivity, and specificity. The predictive performance of the urinary biomarkers and combination models is presented in Table 4, and their ROC curves are presented in Figure 5.

## 4. Discussion

A previous meta-analysis reported that more than one-third of advanced heart failure patients develop AKI after LVAD implantation, and postoperative AKI is associated with significantly increased 30-day and 1-year mortality [27]. If perioperative AKI is recognized early or even predicted preoperatively, nephroprotective measures could be considered to minimize renal insults and potentially avoid the development of postoperative AKI and improve prognosis. This underscores the critical need for reliable biomarkers to identify high-risk patients prior to surgery. In this study, we investigated urine VDBP and KIM-1 in advanced heart failure patients before and after LVAD implantation. Both urinary biomarkers demonstrated strong predictive performance for postoperative AKI when measured preoperatively, with superior sensitivity and specificity compared to their postoperative counterparts. Furthermore, the predictive accuracy of urinary VDBP and KIM-1 improved substantially after adjusting for baseline renal function and hemodynamic status. These findings support the clinical utility of urinary VDBP and KIM-1 as promising non-invasive biomarkers for AKI risk stratification in LVAD recipients.

In one animal model study, urinary VDBP excretion increased early after kidney injury [28]. Clinically, it has been found that excessive excretion of urine VDBP could indicate tubular dysfunction, which is considered an early hallmark of AKI [14,29,30]. Moreover, urinary VDBP level was found to be significantly negatively correlated with eGFR level in various populations [31,32]. In this study, significantly elevated urinary VDBP/creatine was observed in LVAD patients with abnormal kidney function, and we noticed a significant negative correlation between urinary VDBP and eGFR. Urinary VDBP level may help physicians understand these patients’ kidney health before and after LVAD implantation. Moreover, one recent study from Joseph et al. proposed that urinary VDBP can be a strong early prognostic predictor for postoperative AKI in cardiac surgery patients [15]. Similarly, in diabetes mellitus patients with mild renal impairment undergoing coronary angiography, increased urine VDBP concentration 24 h after the procedure was associated with a significantly higher incidence of renal replacement therapy requirement [28]. Based on these findings, urine VDBP has been evaluated in many clinical cohorts of AKI, including post-cardiac surgery AKI, although no clinical study has evaluated the performance of urine VDBP in LVAD patients. We report for the first time our demonstration that urine VDBP is significantly associated with the risk of postoperative AKI in LVAD patients.

It was shown in various AKI animal models that KIM-1 expression was upregulated on the apical surface of the epithelial cells of the renal proximal tubules [33,34]. The ectodomain of KIM-1 is cleaved by matrix metalloproteinases and is present in the urine after kidney proximal tubular injury [34,35]. Building on this, KIM-1 has been proposed as a sensitive and specific non-invasive biomarker of kidney injury in several settings. In one prospective study, KIM-1 level was found to be associated with the severity of renal injury at diagnosis, and elevated KIM-1 levels can increase the risk of AKI after diagnosis in antineutrophil cytoplasmic antibodies-associated vasculitis with glomerulonephritis patients [36]. Moreover, one systematic analysis included a total of 14 studies with 3300 patients to evaluate the predictive ability of urinary KIM-1 for AKI in adult patients; urinary KIM-1 showed high sensitivity and specificity for AKI prediction [37]. Plasma KIM-1 was previously measured in 16 LVAD patients from Liza’s study. However, they did not find a significant correlation between KIM-1 and eGFR, and KIM-1 was not able to predict AKI in LVAD patients [38]. In our study, we observed a significant negative correlation between urine KIM-1 and eGFR. KIM-1 showed good performance for postoperative AKI prediction in LVAD patients. Unlike Liza’s study, we measured KIM-1 from patients’ urine and normalized it with urine creatinine, which may better reflect proximal tubular injury and account for urine concentration variability. This methodological difference may explain the improved predictive performance observed in our study and supports the potential of urinary KIM-1/creatinine ratio as a reliable biomarker for renal risk stratification in the LVAD population.

Intraoperative records revealed no episodes of hypotension or use of nephrotoxic agents in any patient, and CPB duration was comparable between AKI and no-AKI groups, suggesting that intraoperative confounding factors are unlikely to account for the biomarker differences observed. Rather, postoperative AKI in LVAD patients likely reflects hemodynamic alterations after device implantation and preoperative kidney health status. Patients with advanced heart failure who present with impaired renal function and hemodynamic instability appear to be at increased risk for developing postoperative AKI [6]. One retrospective observational study demonstrated that intraoperative hemodynamic disturbances during cardiac surgery are strongly associated with postoperative AKI [39]. Similarly, pre-existing kidney dysfunction at the time of LVAD implantation has been linked to poor renal outcomes following surgery [40]. These findings suggest that both renal and hemodynamic status prior to LVAD implantation may play a pivotal role in the development of postoperative AKI. In our study, we observed distinct renal function trajectories among the four patient groups stratified by pre-LVAD kidney function and hemodynamic stability. While eGFR initially improved during the first postoperative week in all groups, a marked decline was noted by postoperative week two in patients from Groups 3 and 4, who had abnormal renal function and/or unstable hemodynamics. These trends highlight the importance of baseline clinical status in influencing postoperative kidney outcomes. After adjusting for pre-LVAD renal function and hemodynamic status, the association between urinary biomarkers and postoperative AKI was significantly strengthened. Predictive performance also improved when these clinical risk factors were incorporated into biomarker-based models. These findings underscore the clinical value of a combined assessment approach. Preoperative evaluation of kidney function, hemodynamic status, and urinary biomarkers such as VDBP and KIM-1 may provide a comprehensive risk stratification strategy for anticipating postoperative AKI in advanced heart failure patients undergoing LVAD implantation.

Several biomarkers have been investigated for the early detection of AKI following LVAD implantation. In one study, Alam reported that urinary cell-cycle arrest biomarkers insulin-like growth factor-binding protein 7 (IGFBP7) and TIMP-2 can be effective in predicting AKI in LVAD patients [41]. Moreover, Sumida proposed that measurement of perioperative plasma NGAL is useful for predicting severe AKI requiring renal replacement therapy and renal recovery in LVAD patients [10]. However, these studies have very limited sample sizes, and larger studies are still warranted to validate these findings. In this study, we only investigate two kidney damage markers for AKI prediction and cannot compare the prediction performances of these biomarkers with other reported AKI prediction markers, such as NGAL and TIMP2. However, one recent study from Lima et al. has emphasized that integrating structural and functional tubular biomarkers can enhance diagnostic accuracy and provide mechanistic insight into AKI pathophysiology [42]. Consistent with these concepts, our data show that elevated urinary KIM-1 and VDBP levels identify patients with subclinical tubular stress even before overt renal dysfunction, underscoring their translational value for early AKI risk stratification in LVAD recipients. In our previous study, we proposed that the pre-LVAD plasma level of endostatin can be a good biomarker to predict renal function improvement, while the postoperative 1-week plasma level of cystatin C can help early identify high-risk AKI patients in the first month after LVAD implantation [4]. Compared to blood, urine provides an ideal, non-invasive matrix for biomarker assessment, especially for renal diseases whose direct origin is from the kidneys, and it is easy to collect. In this study, we highlight the utility of urinary VDBP and KIM-1 as promising non-invasive biomarkers for predicting postoperative AKI in LVAD patients. Furthermore, we expanded our analysis to evaluate how pre-LVAD kidney function and hemodynamic status influence biomarker performance and AKI risk. By integrating urinary biomarkers with baseline clinical characteristics, we demonstrated enhanced predictive accuracy for AKI, offering a practical and safer alternative to blood sampling in this high-risk population.

## 5. Conclusions

Our study has shown that non-invasive assessment of urinary VDBP and KIM-1 can offer valuable insights into kidney health in LVAD patients, beyond what is gathered from serum creatinine-based and hemodynamic instability-based evaluations. These markers can help predict the potential AKI over a period of 90 days after LVAD implantation.

## 6. Limitations

There are several limitations in our study. Firstly, this is a single-center prospective study with a small sample size. Despite the direction and magnitude of our results being consistent with prior larger studies in cardiac surgery and heart failure populations [14,15,18,37,43], supporting the biological plausibility of our findings, they should be interpreted as exploratory. The limited cohort increases the risk of type I error, and larger multicenter studies are warranted to validate these associations. Second, although intraoperative data revealed no episodes of hypotension or exposure to common nephrotoxic drugs during the surgery, and CPB duration was comparable between AKI and no-AKI groups, other unmeasured perioperative factors, such as fluid balance, could still contribute to postoperative renal outcomes, and confounding from preexisting renal vulnerability cannot be completely excluded. The independent predictive value of urinary VDBP and KIM-1 should be interpreted with caution. Finally, we only measured urinary VDBP and KIM-1 at two time points (pre-LVAD and one week post-LVAD), which may not fully capture the dynamic changes in VDBP and KIM-1 throughout the perioperative and recovery period. Serial measurements at additional time points could provide a more comprehensive understanding.

## Figures and Tables

**Table 1:** Demographic Comparison of LVAD Patients Based on Pre-LVAD Kidney Dysfunction and Hemodynamic Instability.

Characteristics	Group 1 (Normal, Unstable) (N = 7)	Group 2 (Normal, Stable) (N = 10)	Group 3 (Abnormal, Unstable) (N = 6)	Group 4 (Abnormal, Stable) (N = 6)	*p*-Value
Demography					
Age in years, Median (IQR)	59 (41–65)	61 (56–68)	62 (54–64)	64 (49–72)	0.576
Sex, n (% male)	6 (85.71%)	7 (70.00%)	5 (83.33%)	6 (100%)	0.639
Race					0.676
Caucasian white, n (%)	5 (71.43%)	5 (50.00%)	5 (83.33%)	3 (50.00%)	
African American, n (%)	1 (14.29%)	4 (40.00%)	1 (16.67%)	3 (50.00%)	
Other, n (%)	1 (14.29%)	1 (10.00%)	0	0	
Height in meters, Median (IQR)	1.73 (1.68–1.78)	1.69 (1.63–1.75)	1.79 (1.73–1.83)	1.79 (1.73–1.83)	0.173
Weight in kilograms, Median (IQR)	79.9 (67.0–83.6)	78.6 (69.4–95.3)	86.4 (77.7–91.9)	88.1 (75.1–101.5)	0.584
BMI, kg/m^2^, Median (IQR)	25.62 (21.42–28.43)	26.61 (23.56–30.75)	26.58 (23.56–29.44)	26.38 (26.10–29.50)	0.798
BSA, m2, Median (IQR)	1.96 (1.75–2.06)	1.95 (1.75–2.17)	2.17 (1.97–2.24)	2.06 (1.88–2.35)	0.308
History of smoking, n (%)	4 (57.14%)	3 (30.00%)	4 (66.67%)	2 (33.33%)	0.459
History of alcohol abuse, n (%)	3 (42.86%)	4 (40.00%)	2 (33.33%)	3 (50.00%)	1.000
History of drug abuse, n (%)	1 (14.29%)	0	1 (16.67%)	0	0.421
Hypertension, n (%)	2 (28.57%)	7 (70.00%)	3 (50.00%)	4 (66.67%)	0.382
Diabetes, n (%)	4 (57.14%)	4 (40.00%)	1 (16.67%)	2 (33.33%)	0.615
COPD, n (%)	0	0	1 (16.67%)	1 (16.67%)	0.214
Peripheral vascular disease, n (%)	0	0	2 (33.33%)	1 (16.67%)	0.070
Cerebral vascular accident, n (%)	0	0	0	1 (16.67%)	0.585
SBP (mmHg), Median (IQR)	102 (94–111)	105 (99–115)	103 (88–117)	113 (110–116)	0.547
DBP (mmHg), Median (IQR)	72 (64–72)	72 (59–86)	69 (63–72)	71 (63–77)	0.942
Etiology of heart disease					0.556
Ischemic cardiomyopathy, n (%)	2 (28.57%)	6 (60.00%)	3(50.00%)	4(66.67%)	
Non-ischemic cardiomyopathy, n (%)	5 (71.43%)	4 (40.00%)	3(50.00%)	2(33.33%)	
INTERMACS profile, median (IQR)	2.00 (2.00–2.00)	3 (3.00–3.00)	1.83 (2.00–2.00)	3.00 (3.00–3.00)	<0.001 *
NYHA classification, median (IQR)	3.86 (4.00–4.00)	3.80 (4.00–4.00)	3.83 (4.00–4.00)	4.00 (4.00–4.00)	0.734
Echocardiographic parameters					
LviDd in centimeters, n (%)	6.10 (5.81–6.39)	6.76 (6.33–7.01)	7.09 (6.90–7.32)	6.72 (6.34–6.85)	0.0152 *
LVEF (%)	18.81 (16.90–21.00)	19.46 (17.30–21.90)	20.98 (16.00–24.10)	20.50 (13.40–23.70)	0.811
LVAD implantation goal					0.414
BTT, n (%)	0	0	0	1 (16.67%)	
DT, n (%)	7 (100.00%)	10 (100.00%)	6 (100.00%)	5 (83.33%)	
Post-LVAD mechanical ventilation (h), median (IQR)	63 (40–68)	59 (29–86)	81 (21–69)	77 (25–52)	0.756
Post-LVAD ICU stay (days), Median (IQR)	15 (9–16)	11 (7–15)	29 (23–34)	18 (10–27)	0.056
Length of total hospitalization (days), Median (IQR)	41 (16–46)	23 (18–23)	48 (24–55)	43 (31–52)	0.043 *

Note: Continuous variables are presented as median with interquartile range, while categorical variables are presented as number and percentage. Statistical analysis comparing the groups was performed using non-parametric One-Way analysis of variance (ANOVA) with Kruskal-Wallis Test for continuous variables and Chi-square test for categorical variables. * *p* < 0.05 is considered statistically significant. BMI, body mass index; BSA, body surface area; BTT, bridge to transplantation; COPD, chronic obstructive pulmonary disease; DBP, diastolic blood pressure; DT, destination therapy; ICU; intensive care unit; INTERMACS, Interagency Registry for Mechanically Assisted Circulatory Support; LVEF, left ventricular ejection fraction; LviDd, left ventricular internal diameter at end-diastole; NYHA, New York Heart Association; SBP, systolic blood pressure.

**Table 2:** Comparison of Demographics in LVAD Patients: Post-LVAD AKI vs. No AKI Within 90 Days Post-Surgery.

Characteristics	No-AKI (N = 16)	AKI (N = 13)	*p*-Value
Demography			
Age in years, Median (IQR)	62 (49–66)	61 (54–67)	0.948
Sex, n (% male)	14 (87.50%)	10 (76.92%)	0.632
Race			0.168
Caucasian white, n (%)	11 (68.75%)	7 (53.85%)	
African American, n (%)	3 (18.75%)	6 (46.15%)	
Other, n (%)	2 (12.50%)	0	
Height in meters, Median (IQR)	1.73 (1.65–1.79)	1.74 (1.70–1.83)	0.322
Weight in kilograms, Median (IQR)	80.5 (72.3–86.1)	88.2 (73.5–96.6)	0.273
BMI, kg/m^2^, Median (IQR)	25.9 (22.9–30.1)	26.4 (25.5–29.4)	0.776
BSA, m^2^, Median (IQR)	1.97 (1.77–2.09)	2.11 (1.86–2.24)	0.254
History of smoking, n (%)	8 (50.00%)	5 (38.46%)	0.711
History of alcohol abuse, n (%)	6 (37.50%)	6 (46.15%)	0.716
History of drug abuse, n (%)	1 (6.25%)	1 (7.69%)	1.000
Hypertension, n (%)	8 (50.00%)	8 (61.54%)	0.711
Diabetes, n (%)	7 (43.75%)	4 (30.77%)	0.702
COPD, n (%)	1 (6.25%)	1 (7.69%)	1.000
CKD, n (%)	11 (68.75%)	10 (76.92%)	0.697
Peripheral vascular disease, n (%)	2 (12.50%)	1 (7.69%)	1.000
Cerebral vascular accident, n (%)	2 (12.50%)	1 (7.69%)	1.000
SBP (mmHg), Median (IQR)	102 (93–112)	109 (104–116)	0.046
DBP (mmHg), Median (IQR)	67 (59–73)	74 (67–82)	0.865
Etiology of heart disease			0.715
Ischemic cardiomyopathy, n (%)	9 (56.25%)	7 (43.75%)	
Non-ischemic cardiomyopathy, n (%)	6 (46.15%)	7 (53.85%)	
INTERMACS profile, median (IQR)	2.38 (2.00–3.00)	2.69 (3.00–3.00)	0.071
NYHA classification, median (IQR)	4 (4–4)	4 (4–4)	0.826
Echocardiographic parameters			
LviDd in centimeters, n (%)	6.54 (6.32–6.90)	6.80 (6.34–7.01)	0.469
LVEF (%)	20.71 (18.05–23.90)	18.76 (16.90–21.90)	0.148
LVAD implantation goal			0.448
BTT, n (%)	0	1 (7.69%)	
DT, n (%)	16 (100.00%)	12 (92.31%)	
Length of CPB (min), median (IQR)	97 (69–105)	113 (59–132)	0.568
Post-LVAD mechanical ventilation (h), median (IQR)	46 (22–52)	103 (40–140)	0.064
Post-LVAD ICU stay (days), Median (IQR)	16 (8–20)	20 (11–27)	0.195
Length of total hospitalization (days), Median (IQR)	34 (16–39)	47 (31–52)	0.012 *

Note: Continuous variables are presented as median with interquartile range, while categorical variables are presented as number and percentage. Statistical analysis comparing the groups was performed using non-parametric One-Way analysis of variance (ANOVA) with Kruskal-Wallis Test for continuous variables and Chi-square test for categorical variables. * *p* < 0.05 is considered statistically significant. BMI, body mass index; BSA, body surface area; BTT, bridge to transplantation; COPD, chronic obstructive pulmonary disease; CPB, cardiopulmonary bypass; CKD, chronic kidney disease; DBP, diastolic blood pressure; DT, destination therapy; ICU; intensive care unit; INTERMACS, Interagency Registry for Mechanically Assisted Circulatory Support; LVEF, left ventricular ejection fraction; LviDd, left ventricular internal diameter at end-diastole; NYHA, New York Heart Association; SBP, systolic blood pressure.

**Table 3:** Odds ratio for urinary VDBP and KIM-1 with AKI.

	Odds Ratio (95% Confidence Intervals)		
	Unadjusted	Model 1	Model 2
Pre-LVAD: uKIM1			
Per two-fold greater uKIM1	1.98 (1.28–3.71)	4.39 (1.79–22.2)	8.76 (2.03–311)
Pre-LVAD: uVDBP			
Per two-fold greater uVDBP	1.99 (1.27–3.68)	2.25 (1.23–4.90)	7.74 (2.07–296)
Post-LVAD: uKIM1			
Per two-fold greater uKIM1	1.39 (0.84–2.30)	1.59 (0.86–2.94)	1.65 (0.87–3.16)
Post-LAVD: uVDBP			
Per two-fold greater uVDBP	1.26 (0.77–2.08)	1.20 (0.71–2.03)	1.01 (0.56–1.84)
Model 1 was adjusted for pre-LVAD kidney dysfunction. Model 2 was adjusted for pre-LVAD kidney dysfunction and baseline hemodynamic instability			

Note: AKI, acute kidney injury; uVDBP, urinary Vitamin D binding protein; uKIM-1, urinary kidney injury molecule-1; uCr, urinary creatinine.

**Table 4:** Urinary biomarkers and adjusted models for predicting AKI after LVAD implantation.

Variables and Models		AUC (95%CI)	Sensitivity (%)	Specificity (%)	*p* Values
Pre-LVAD	uKIM-1	0.832 (0.647–0.944)	84.6%	81.2%	<0.001
	uKIM-1/Model 1	0.856 (0.709–0.999)	92.3%	75.0%	<0.001
	uKIM-1/Model 2	0.923 (0.814–0.999)	92.3%	81.2%	<0.001
	uVDBP	0.841 (0.658–0.950)	84.6%	81.2%	<0.001
	uVDBP/Model 1	0.846 (0.695–0.997)	84.6%	81.2%	<0.001
	uVDBP/Model 2	0.952 (0.865–0.999)	92.3%	87.5%	<0.001
Post-LVAD	uKIM-1	0.798 (0.608–0.923)	76.9%	81.2%	0.001
	uKIM-1/Model 1	0.808 (0.641–0.974)	84.6%	75.0%	<0.001
	uKIM-1/Model 2	0.851 (0.702–0.999)	76.9%	93.8%	<0.001
	uVDBP	0.798 (0.608–0.923)	76.9%	87.5%	0.002
	uVDBP/Model 1	0.812 (0.648–0.977)	76.9%	93.8%	<0.001
	uVDBP/Model 2	0.832 (0.675–0.989)	84.6%	75.0%	<0.001

Note: AKI, acute kidney injury; uVDBP, urinary Vitamin D binding protein; uKIM-1, urinary kidney injury molecule-1; AUC, area under the receiver operating characteristic; CI, confidence intervals; Model 1 was adjusted for baseline kidney dysfunction; Model 2 was adjusted for baseline kidney dysfunction and baseline hemodynamic instability.

## Data Availability

Due to privacy and ethical restrictions, personal medical data of patients is not publicly available and was obtained from the medical records of patients admitted to our hospital. The original contributions presented in this study are included in the article/supplementary material. De-identified raw data supporting the findings are available from the corresponding author upon reasonable request.

## Supplementary Materials

The following supporting information can be downloaded at: https://www.mdpi.com/article/10.3390/biomedicines13112682/s1, Table S1: Summary of changes in renal function and urinary biomarker levels in the four patient groups; Table S2: Summary of p-values in renal function and urinary biomarker levels in the four patient groups from Table S1; Table S3: Summary of changes in renal function and urinary biomarker levels in AKI and no-AKI group patients.
