# Supplementary material for: Urinary Vitamin D Binding Protein and Kidney Injury Molecule-1 Are Potent Predictors of Acute Kidney Injury After Left Ventricular Assist Device Implantation
Source: Biomedicines. 2025 Oct 31;13(11):2682. doi: 10.3390/biomedicines13112682 (PMC12650049; doi:10.3390/biomedicines13112682)
Supplement: Supplementary file 1 [file biomedicines-13-02682-s001.zip › biomedicines-3914624-supplementary.pdf]

Online Supplement

Table S1. Summary of changes in renal function and urinary biomarker levels in the four patient groups.

**Variables**	**Group 1 (normal, unstable) (n=7)**	**Group 2 (normal, stable) (n=10)**	**Group 3 (abnormal, unstable) (n=6)**	**Group 4 (abnormal, stable) (n=6)**
BUN (mg/dl), Mean ± sd				
Base	19.286 ± *5.122*	17.250 ± *5.258*	40.333 ± *29.310*	41.833 ± *17.509*
POD7	21.000 ± *10.198*	27.375 ± *22.652*	20.833 ± *7.731*	39.800 ± *10.257*
POD14	16.667 ± *8.311*	25.111 ± *19.946*	31.167 ± *27.622*	78.200 ± *28.128*
POD21	18.000 ± *8.198*	18.500 ± *11.464*	31.500 ± *22.554*	41.800 ± *33.260*
POD30	14.500 ± *10.840*	16.500 ± *5.976*	26.000 ± *11.045*	44.333 ± *26.204*
POD60	15.286 ± *6.897*	15.000 ± *5.518*	27.000 ± *5.727*	29.167 ± *17.860*
POD90	19.000 ± *10.826*	17.100 ± *7.280*	23.000 ± *6.976*	22.500 ± *5.357*
Creatinine (mg/dl), Mean ± sd				
Base	0.981 ± *0.234*	0.976 ± *0.185*	1.533 ± *0.189*	1.953 ± *0.376*
POD7	0.880 ± *0.303*	1.025 ± *0.614*	0.980 ± *0.332*	1.598 ± *0.410*
POD14	0.847 ± *0.371*	1.071 ± *0.615*	1.230 ± *0.307*	2.544 ± *1.308*
POD21	0.970 ± *0.532*	1.006 ± *0.463*	1.190 ± *0.182*	1.464 ± *0.792*
POD30	1.000 ± *0.490*	1.136 ± *0.486*	1.312 ± *0.501*	1.323 ± *0.370*
POD60	1.094 ± *0.436*	0.983 ± *0.447*	1.382 ± *0.404*	1.512 ± *0.677*
POD90	1.323 ± *0.594*	1.034 ± *0.354*	1.270 ± *0.444*	1.397 ± *0.474*
eGFR, Mean ± sd				
Base	87.857 ± *19.187*	77.500 ± *18.131*	46.833 ± *6.794*	41.167 ± *6.998*
POD7	102.143 ± *30.981*	82.900 ± *31.596*	84.167 ± *24.482*	54.833 ± *15.459*
POD14	100.714 ± *25.051*	80.100 ± *35.874*	66.833 ± *23.845*	37.833 ± *14.442*
POD21	92.857 ± *32.251*	81.300 ± *30.689*	65.667 ± *13.456*	68.333 ± *25.216*
POD30	92.000 ± *26.981*	73.100 ± *34.070*	59.333 ± *15.693*	67.333 ± *17.974*
POD60	81.571 ± *24.986*	85.556 ± *30.851*	59.333 ± *17.489*	63.333 ± *25.430*
POD90	69.500 ± *28.438*	82.000 ± *24.708*	65.6750 ± *16.601*	65.833 ± *23.794*
Pre-LVAD				
uVDBP/uCr (ng/mg), Median (IQR)	0.241(0.192-1.253)	0.254(0.179-1.052)	3.807(0.879-6.973)	1.794(0.221-3.102)
uKIM-1/uCr (ng/mg), Median (IQR)	0.002(0.001-0.021)	0.011(0.004-0.018)	0.057(0.012-0.104)	0.059(0.013-0.097)
Post-LVAD				
uVDBP/uCr (ng/mg), Median (IQR)	0.310(0.207-1.901)	3.769(0.379-8.516)	2.113(0.864-5.380)	4.330(2.857-7.124)
uKIM-1/uCr (ng/mg), Median (IQR)	0.005(0.004-0.020)	0.025(0.007-0.064)	0.068(0.019-0.140)	0.140(0.024-0.236)

Note: Routine laboratory kidney function profile variables (BUN, creatinine, and eGFR) are presented as mean ± standard deviation (sd), urinary markers uVDBP/uCr and uKIM-1/uCr are presented as median with IQR. BUN, blood urea nitrogen; eGFR, estimated glomerular filtration rate; POD; post-operative day; uVDBP, urinary Vitamin D binding protein; uKIM-1, urinary kidney injury molecule-1; uCr, urinary creatinine

Table S2. Summary of p-values in renal function and urinary biomarker levels in the four patient groups from Table S1.

Variables	(Gr.1 vs. Gr.2) p-value	(Gr.1 vs. Gr.3) p-value	(Gr.1 vs. Gr.4) p-value	(Gr.2 vs. Gr.3) p-value	(Gr.2 vs. Gr.4) p-value	(Gr.3 vs. Gr.4) p-value
BUN (mg/dl), Mean ± sd						
Base	0.463	0.087	*0.008	*0.047	*0.003	0.916
POD7	0.506	0.975	*0.011	0.514	*0.038	*0.007
POD14	0.349	0.246	*0.001	0.629	*0.001	*0.021
POD21	0.929	0.198	0.122	0.182	0.091	0.556
POD30	0.665	0.099	*0.028	0.060	*0.012	0.145
POD60	0.926	0.227	0.383	0.401	0.232	0.783
POD90	0.679	0.535	0.494	0.192	0.139	0.901
Creatinine (mg/dl), Mean ± std						
Base	0.963	*0.001	*<0.001	*<0.001	*<0.001	*0.035
POD7	0.582	0.582	*0.006	0.874	*0.044	*0.022
POD14	0.440	0.080	*0.014	0.571	*0.013	*0.040
POD21	0.894	0.361	0.248	0.379	0.210	0.428
POD30	0.614	0.301	0.226	0.522	0.448	0.964
POD60	0.618	0.247	0.207	0.096	0.079	0.695
POD90	0.238	0.883	0.818	0.313	0.102	0.683
eGFR, Mean ± sd						
Base	0.275	*<0.001	*<0.001	*0.002	*<0.001	*0.185
POD7	0.232	0.276	*0.006	0.934	*0.044	*0.033
POD14	0.211	*0.030	*<0.001	0.437	*0.016	*0.029
POD21	0.466	0.082	0.160	0.262	0.399	0.824
POD30	0.241	*0.025	0.084	0.372	0.709	0.431
POD60	0.785	0.095	0.220	0.083	0.168	0.757
POD90	0.382	0.820	0.814	0.260	0.230	0.995
Pre-LVAD						
uVDBP/uCr (ng/mg), Median (IQR)	0.604	0.062	0.114	*0.008	*0.001	0.276
uKIM-1/uCr (ng/mg), Median (IQR)	0.439	0.059	*0.017	*0.032	*0.009	0.544
Post-LVAD						
uVDBP/uCr (ng/mg), Median (IQR)	0.171	0.157	*0.019	0.152	0.453	0.976
uKIM-1/uCr (ng/mg), Median (IQR)	0.118	0.259	*0.021	0.453	*0.038	0.416

Note: Statistical analysis comparing the groups (Gr.) was performed using the Mann-Whitney U test. $p<0.05$ is considered statistically significant. ANOVA, analysis of variance; BUN, blood urea nitrogen; eGFR, estimated glomerular filtration rate; POD, post-operative day; uVDBP, urinary Vitamin D binding protein; uKIM-1, urinary kidney injury molecule-1; uCr, urinary creatinine.

Table S3. Summary of changes in renal function and urinary biomarker levels in AKI and no-AKI group patients

**Characteristics**	**No-AKI (N=16)**	**AKI (N=13)**	**p-value**
BUN (mg/dl), Mean ± sd			
Base	23.643 ± *9.311*	30.857 ± *31.662*	0.709
POD7	24.308 ± *13.300*	29.857 ± *24.674*	0.905
POD14	22.583 ± *11.813*	49.875 ± *37.567*	*0.049
POD21	20.091 ± *8.826*	40.875 ± *31.165*	*0.039
POD30	21.333 ± *13.799*	37.125 ± *18.271*	*0.034
POD60	19.813 ± *10.722*	28.231 ± *14.709*	0.119
POD90	19.308 ± *9.223*	25.462 ± *11.333*	0.173
Creatinine (mg/dl), Mean ± sd			
Base	1.263 ± *0.383*	1.054 ± *0.410*	0.117
POD7	0.999 ± *0.396*	1.344 ± *0.578*	0.166
POD14	0.928 ± *0.251*	1.813 ± *1.383*	*0.045
POD21	1.025 ± *0.399*	1.624 ± *0.625*	*0.019
POD30	1.112 ± *0.481*	1.666 ± *0.654*	*0.029
POD60	1.162 ± *0.444*	1.482 ± *0.572*	0.100
POD90	1.220 ± *0.502*	1.375 ± *0.424*	0.329
eGFR, Mean ± sd			
Base	67.857 ± *21.750*	76.778 ± *27.266*	0.219
POD7	89.000 ± *33.266*	73.000 ± *34.271*	0.257
POD14	89.786 ± *22.888*	54.333 ± *34.760*	*0.010
POD21	86.000 ± *24.816*	58.222 ± *20.566*	*0.017
POD30	83.857 ± *29.555*	67.556 ± *15.828*	0.123
POD60	77.533 ± *29.181*	70.308 ± *24.854*	0.596
POD90	71.750 ± *25.169*	73.231 ± *24.011*	0.807
Pre-LVAD			
uVDBP/uCr (ng/mg), Median (IQR)	0.231(0.179-0.867)	2.349(1.052-3.582)	*0.033
uKIM-1/uCr (ng/mg), Median (IQR)	0.006(0.002-0.126)	0.042(0.018-0.092)	*0.014
Post-LVAD			
uVDBP/uCr (ng/mg), Median (IQR)	1.445(0.296-2.912)	5.380(3.327-8.516)	*0.027
uKIM-1/uCr (ng/mg), Median (IQR)	0.011(0.004-0.024)	0.064(0.027-0.224)	*0.006

Note: Routine laboratory kidney function profile variables (BUN, creatinine and eGFR) are presented as mean ± standard deviation (sd), urinary markers uVDBP/uCr and uKIM-1/uCr are presented as median with IQR. Statistical analysis comparing the groups was performed using Mann-Whitney U test. $p<0.05$ considered statistically significant. ANOVA, analysis of variance; BUN, blood urea nitrogen; eGFR, estimated glomerular filtration rate; POD; post-operative day; uVDBP, urinary Vitamin D binding protein; uKIM-1, urinary kidney injury molecule-1; uCr, urinary creatinine.
